# High frequency direct shoot regeneration from Kazakh commercial potato cultivars

**DOI:** 10.7717/peerj.9447

**Published:** 2020-07-13

**Authors:** Laura S. Abeuova, Balnur R. Kali, Aizhan O. Rakhimzhanova, Sara S. Bekkuzhina, Shuga A. Manabayeva

**Affiliations:** 1 Plant Genetic Engineering Laboratory, National Center for Biotechnology, Nur-Sultan, Kazakhstan; 2 Faculty of Natural Sciences, L.N.Gumilyov Eurasian National University, Nur-Sultan, Kazakhstan

**Keywords:** *Solanum tuberosum* L., Tissue culture, Direct regeneration, Plant growth regulator, Microtuber

## Abstract

Potato (*Solanum tuberosum* L.) is the third most economically important crop in the world and has a high nutritional value. In this study, the in vitro culture response of four widely grown in Kazakhstan potato cultivars, Astanalyk, Monument Kunaev, Tokhtar, and Aksor, was investigated using stem and leaf explants. Published protocols were evaluated and optimized to develop a more efficient protocol for the regeneration of plants from local potato cultivars in tissue culture, which is a prerequisite to facilitate potato genome modification. The explants were cultured on solid Murashige and Skoog medium supplemented with different concentrations and combinations of zeatin, 6-benzylaminopurine (BAP), gibberellic acid (GA_3_), 1-naphthaleneacetic acid (NAA) and indole-3-acetic acid (IAA). The maximum regeneration was induced from the stem internodal explants. A significant effect of the explant source on direct regeneration was confirmed with statistical analysis. The number of shoots obtained from the internode was 10.0 from cv. Aksor followed by cvs. Tokhtar and Astanalyk. The medium DRM-VIII with 1 mg/l zeatin, 0.1 mg/l IAA and 7.0 mg/l GA_3_ was considered the best for direct shoot regeneration and multiple shoot formation from all cultivars. To conclude, we outline a protocol for direct plant regeneration from four potato cultivars. Our findings suggest commercial cultivars Astanalyk and Aksor are good candidates for developing the genome-edited plants through direct shoot regeneration.

## Introduction

Potato (*Solanum tuberosum* L.) is the third most economically important food crop worldwide, exceeded only by wheat and rice (Birch et al., 2012), and is an important constituent in human diets. Consequently, potato is widely used in food-processing industries. Products containing processed potatoes include starch, frozen French fries, chips, and dehydrated potatoes. Annual worldwide potato production is approximately 330 million tons (Monneveux, Ramírez & Pino, 2013), and Kazakhstan is producing annually around 2.5 million tons from an area of 240,000 ha. with an average annual per capita consumption of up to 100 kg. Since potato production has a strong economic and social importance, it has been the subject of numerous studies also in the field of plant biotechnology. The success of potato biotechnology relies on several factors, including an efficient tissue culture system for the regeneration of plants from cultured cells and tissues. Many studies have reported that often most of the tissue culture process is cultivar-specific. It is, therefore, necessary to establish a specific protocol for each genotype studied (Campos et al., 2016; Tara, Krishnaprasad & Veena, 2017; Yadav & Sticklen, 1995).

The success of potato in vitro culture depends on the explant, culture medium, and combination and concentration of plant growth regulators (PGRs) in the culture medium (Afrasiab, Rashid & Akram, 2017; AL-Hussaini, Yousif & AL-Ajeely, 2015; Kumlay, 2014; Mohapatra et al., 2018). Shoot culture is the basic technique of potato biotechnology. Meristem tip culture of potatoes is commonly used for the production of virus-free potato plants, and for mass propagation it can be used in Murashige and Skoog (MS) medium without exogenous PGRs, although it benefits from the addition of some PGRs at very low concentrations (Miller et al., 1985). Direct and indirect regeneration of potato plants has been induced in various in vitro cultured explants by the external addition of PGRs (Jones, Karp & Jones, 1989; Tara, Krishnaprasad & Veena, 2017), and large variations in the efficiency of shoot formation among commercial potatoes cultivars have been noted. Efficient direct and indirect regeneration protocols have been demonstrated for cultivar Kufri Jyoti (Manisha & Tapan, 2015) in which IAA and GA_3_ are optimal PGRs for direct regeneration through node explants. The effect of PGRs on direct and indirect regeneration of potato cultivar Kufri Jyoti from internodes and leaf explants has also been investigated by other groups, who found that the plants regenerated by being placed on MS medium supplemented with 3 mg/l BAP and 1 mg/l GA_3_ (Tara, Krishnaprasad & Veena, 2017). in vitro responses for another three potato cultivars studied using diverse explants (Hussain et al., 2005) showed that the nodal tissues are more responsive to direct regeneration than the shoot apices, leaf discs, and intermodal explants on media supplemented with BAP and IAA at concentrations of 2.0 and 0.5 mg/l, respectively. Callus formation and organogenesis have been studied for potato cultivar Almera, and TDZ at a concentration of 5.0 mg/l was shown to be the most active cytokinin for plant regeneration (Mutasim, Khadiga & Rasheid, 2010). A specific regeneration protocol has been established for cultivar Monalisa, and the best PGR combinations for shoot regeneration from potato internodes were considered to be zeatin, NAA, and GA_3_ at concentrations of 3 mg/l, 0.05 mg/l, and 0.10 mg/l, respectively (Campos et al., 2016). An effective protocol has also been developed for cultivars Arnova, Burren, Provento, and Riviera (AL-Hussaini, Yousif & AL-Ajeely, 2015). The best results for callus induction and regeneration were obtained from MS medium containing 3 mg/l BAP, 0.03 mg/l NAA and 0.5 mg/l GA_3_ for cultivars Burren, Provento and Riviera, while cultivar Arnova regenerated shoots on media containing 0.22 mg/l TDZ and 0.49 mg/l NAA.

We aim to develop a genome editing technology for commercially grown Kazakh potato cultivars by identifying the optimal way to regenerate shoots from different explants. It is well-established that crop plant regeneration ability is tissue- and genotype-dependent. Hence, we compared the regeneration ability of two different explants from four widely grown in Kazakhstan potato cultivars of Astanalyk, Monument Kunaev, Tokhtar, and Aksor. The protocol developed will assist in obtaining genetically improved plants of these potato cultivars.

## Material and Methods

### Plant material

In vitro shoot culture of *S. tuberosum* L*.* cultivars Astanalyk, Kunaev Monument, Tokhtar and Aksor were obtained from the Laboratory of Plant Biotechnology and Breeding (NCB, Nur-Sultan) and the Laboratory of Breeding and Biotechnology (IPBB, Almaty).

### Culture media and conditions

The direct regeneration (DRM), root induction (RIM) and microtuberization (MT) media were based on MS salts and vitamins (Murashige & Skoog, 1962). All tissue culture media were adjusted to pH 5.6 with 1 N KOH, autoclaved at 121 °C for 20 min. PGRs were filter-sterilized by passing through 0.2 µm Millipore filters and added after autoclaving. The cultures were incubated in the growth room at 25 ± 2 °C for a 16 h photoperiod under the fluorescent tube lumps (Osram FLUORA 36W/77, Germany).

### Direct shoot induction

**Figure 1:**
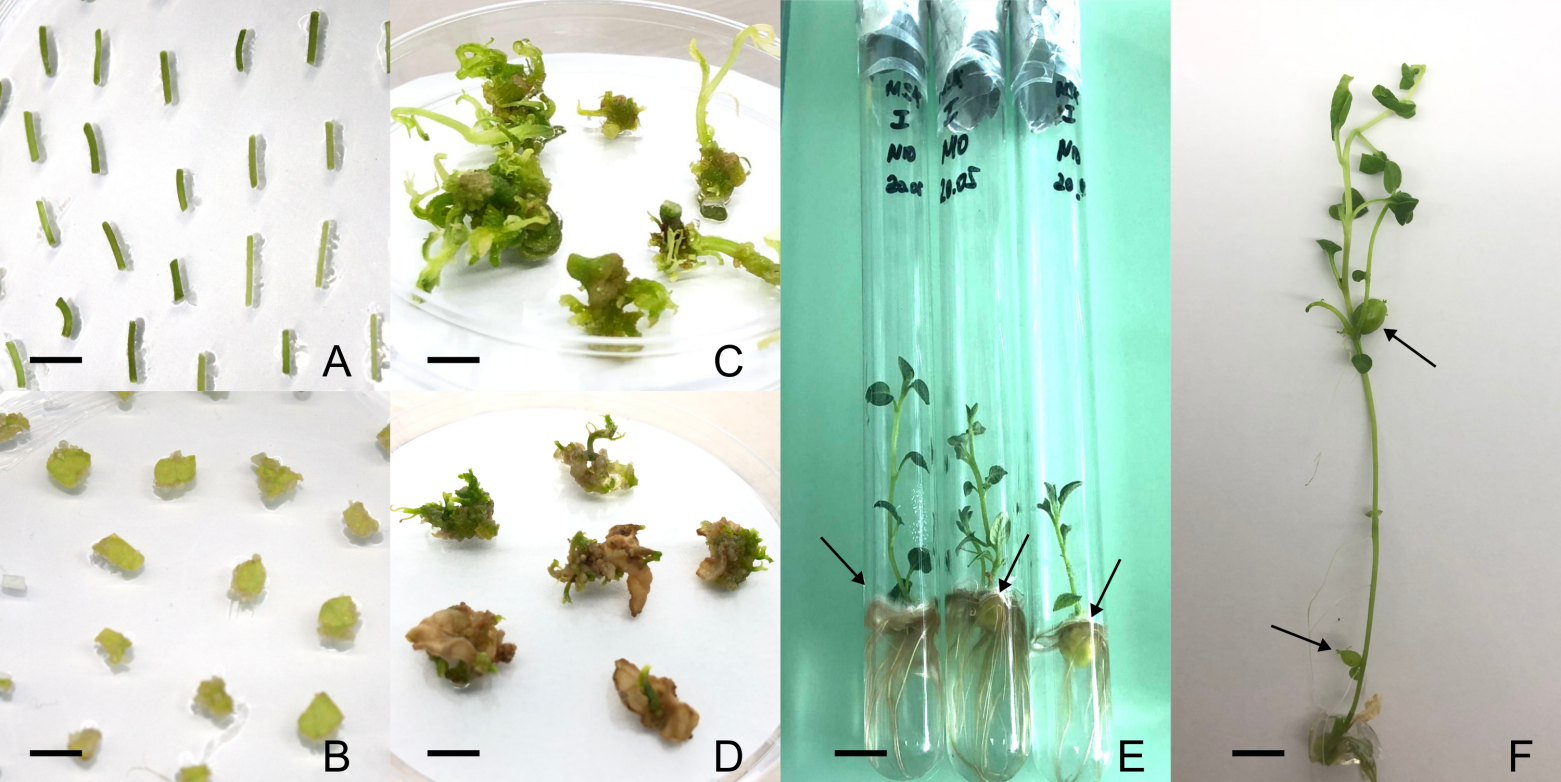
Direct regeneration of potato plants through direct shoot organogenesis (cv. Astanalyk is shown as an example). (A) Stem internode explants. (B) Leaf explants. (C) Shoot induction from internodes. (D) Shoot induction from leaf explants. (E) Induction of green microtubers on axillary buds. (F) Induction of white microtubers on MT-II medium. All scale bars represent 10 mm.

Stem internode (Fig. 1A) and leaf segments (Fig. 1B) obtained from the micro-propagated plantlets on hormone-free MS solid medium (Murashige & Skoog, 1962) containing 3% sucrose, pH 5.8, were used as explants. The leaf and stem internodes were aseptically cut into fragments and cultured in three replicates on the DRM. For direct regeneration, eight different combinations of DRM differing in the concentration of PGRs were used (Table 1). After 4 weeks, the shoot induction data were recorded (Figs. 1C and 1D).

**Table 1:** Treatment descriptions of the different combinations and concentrations of plant growth regulators for direct shoot regeneration.

Medium	Plant grows regulators (mg/l)				
	Zeatin	BAP	GA3	NAA	IAA
DRM-I	0.5		3.0	0.5	
DRM-II	1.0		5.0	0.1	
DRM-III	2.0		7.0	0.2	
DRM-IV		1.0	1.0		
DRM-V		2.0	1.0		
DRM-VI	2.0		0.1	0.1	
DRM-VII	2.0		0.1		0.1
DRM-VIII	1.0		7.0		0.1

### Root induction and microtuberization

For root induction, well-developed shoots were excised from the explants and transferred to RIM supplemented with 3 mg/l IBA. The shoots with the well-developed root system were transferred onto MT-I containing IAA (0.5 mg/l) and kinetin (1.0 mg/l), and MT-II containing kinetin (0.5 mg/l) and ascorbic acid (1.0 mg/l) in the presence 5% sucrose. The efficiency of microtuberization was calculated by counting the shoots that formed at least one microtuber out of the total number of shoots cultivated on MT media after 4 weeks.

### Regeneration rate (%)

Regeneration rate (%) of internodal and leaf explants was determined after 4 to 5 weeks of culture. It was calculated using the following formula:

\begin{eqnarray*}\text{Regeneration rate}(\text{%})=\sum \mathrm{Xi/ (N)}\times 100 \end{eqnarray*}

where ∑= Summation

Xi = Number of explants regenerated

N = Number of explants cultured

### Statistical analysis

The experiments were independently repeated three times, and the data represented the mean ± standard deviation (SD) of the replicates. The data were subjected to two-way ANOVA followed by the Bonferroni post hoc test (*p < 0.05) using Prism 5 (GraphPad Software Inc., La Jolla, CA, USA).

## Results

### Direct shoot regeneration

Direct shoot regeneration of the potato cultivars from Kazakhstan breeding was evaluated by using two types of explants. The stem internodes of all cultivars produced shoots on eight different combinations of DRM containing various combinations and concentrations of PGRs (Table 2). It is known that the meristematic section is one of the most important prerequisites for direct shoot regeneration. Significant differences were observed between cultivars and the best medium for direct shoot regeneration was DRM-VIII supplemented with 0.1 mg/l IAA, 1.0 mg/l zeatin, and 7.0 mg/l GA_3_, where cv. Astanalyk gave the highest shoot induction (90.0%), followed by cvs. Aksor (87.5%) and Tokhtar (70.0%). On this medium, the leaf explants of cvs. Aksor and Tokhtar gave the best results by inducing 68% and 30.0% of shoots. All cultivars were able to induce the shoots in DRM by combinations of PGRs NAA, zeatin and GA_3_ at various concentrations. The lowest shoot induction, in cv. Monument Kunaev (2.5%), was observed on DRM-VIII from leaf explants. The cvs. Aksor and Tokhtar, in turn gave the lowest shoot induction (9.0% and 3.7%, respectively) on DRM-IV containing BAP and GA_3_. On the media containing the combination of BAP and GA_3_, the explants failed to induce shoots, e.g., cv. Kunaev Monument on DRM-IV and V.

**Table 2:** Effect of different culture medium on shoot regeneration of local potato cultivars from internodes and leaf explants. Regeneration rate of internode and leaf explants (%). Each value represents mean SD of three replicated experiments.

Medium	Cultivars /explant types									
	Astanalyk		Aksor			Tokhtar			Kunaev Monument	
	internode	leaf	internode	leaf		internode	leaf		internode	leaf
DRM-I	14.8 ± 0.09	5.5 ± 0.03	45.8 ± 0.18	33.9 ± 0.14		18.4 ± 0.09	7.8 ± 0.05		5.5 ± 0.05	0
DRM-II	26.9 ± 0.1	5.0 ± 0.04	37.6 ± 0.16	29.2 ± 0.12		26.9 ± 0.1	11.7 ± 0.06		13.1 ± 0.1	12.5 ± 0.07
DRM-III	21.35 ± 0.1	5.7 ± 0.06	24 ± 0.13	16.7 ± 0.09		17.7 ± 0.1	5.88 ± 0.04		0	0
DRM-IV	3.2 ± 0.03	0	9.0 ± 0.08	0		3.7 ± 0.03	0		0	0
DRM-V	4.38 ± 0.03	0	16.6 ± 0.08	0		10.0 ± 0.07	0		0	0
DRM-VI	24.0 ± 0.1	0	13.6 ± 0.08	0		30.2 ± 0.17	5.0 ± 0.08		5.0 ± 0.05	0
DRM-VII	43.5 ± 0.1	7.1 ± 0.06	47.0 ± 0.16	37.6 ± 0.15		37.4 ± 0.14	6.23 ± 0.05		14.4 ± 0.11	4.1 ± 0.04
DRM-VIII	90.0 ± 0.1	18.5 ± 0.09	87.5 ± 0.1	68.0 ± 0.14		70.0 ± 0.15	30.0 ± 0.12		31.1 ± 0.16	2.5 ± 0.02

### Multiple shoot formation

**Figure 2:**
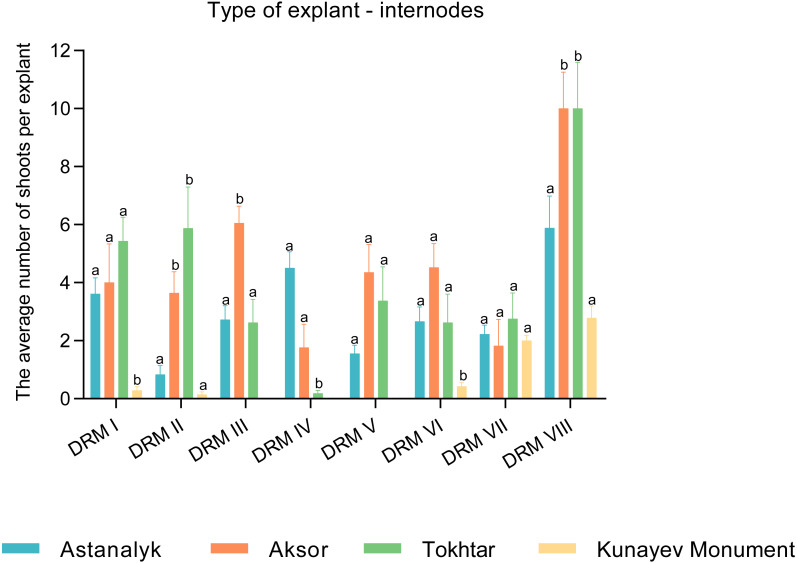
Comparison of regenerated shoots per stem internode explant, pcs. The effects of different growth regulators on multiple shoot induction from the stem internode explants of the cultivars Astanalyk, Kunaev Monument, Tokhtar, and Aksor. The average number of shoots per internode explants was calculated by counting the explants that induced at least one shoot out of the total number of internode explants. The cultivars were compared among themselves in each variant of the medium. Data represent mean ± standard deviation (SD) of three independent experiments with internode explants. Data with the same letters are not significantly different according to two-way ANOVA followed by the Bonferroni post hoc test (^∗^*p* < 0.05) using Prism 5.

**Figure 3:**
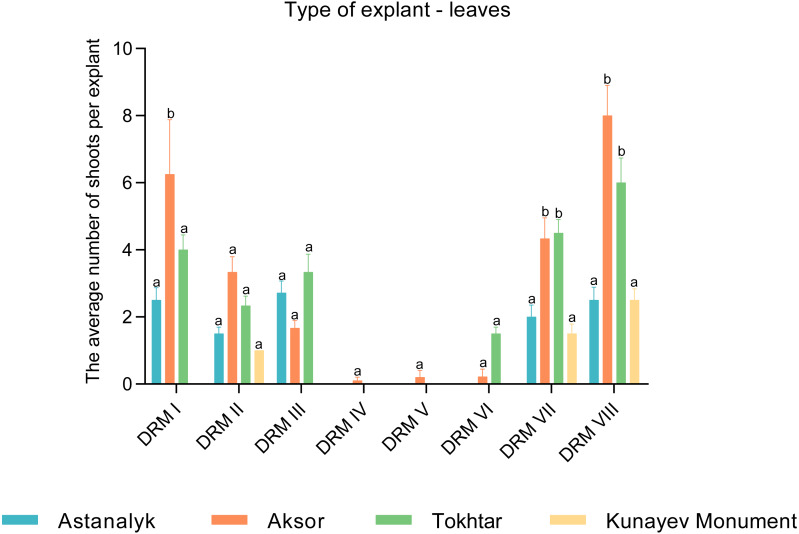
Comparison of regenerated shoots per leaf explant, pcs. The effects of different growth regulators on multiple shoot induction from the leaf explants of the cultivars Astanalyk, Kunaev Monument, Tokhtar, and Aksor. The average number of shoots per leaf explants was calculated by counting the explants that induced at least one shoot out of the total number of leaf explants. The cultivars were compared among themselves in each variant of the medium. Data represent mean ± standard deviation (SD) of three independent experiments with leaf explants. Data with the same letters are not significantly different according to two-way ANOVA followed by the Bonferroni post hoc test (^∗^*p* < 0.05) using Prism 5.

The effects of different growth regulators on multiple shoot induction from the stem internode explants of the cultivars Astanalyk, Kunaev Monument, Tokhtar, and Aksor are shown in Fig. 2. The results demonstrate that all cultivars were able to produce multiple shoots. The maximum number of shoots per explant (21.0) was induced by the internodes of cv. Aksor on the medium DRM-VIII, followed by cv. Tokhtar (19.0) on the same medium. The lowest number of shoots was observed in cv. Kunaev Monument, where 1–5 shoots were induced per explant. A comparison of the effects of different growth regulators on multiple shoot induction from the leaf explants revealed that all cultivars were able to produce multiple shoots on the media DRM-II, DRM-VII, and DRM-VIII (Fig. 3). The best potential of leaf explants was observed when the medium was supplemented with zeatin, IAA, and GA_3_(1.0, 0.1, and 7.0 mg/l, respectively). Our study showed the interaction of specific explants and media yielding the highest number of shoots from internode and leaf explants on the medium DRM-VIII. Overall, the maximum mean was highly significant from the internode explants (10.0) and the leaf explants (8.0) of cv. Aksor.

### Root induction and microtuberization

The cultivars Aksor and Tokhtar induced green microtubers on axillary buds during micropropagation on a hormone-free MS medium containing 3% sucrose. The plantlets regenerated from the internode explants of cv. Aksor also formed microtubers on the axillary buds under light on the medium DRM-VIII (Fig. 1E).

For microtuberization one-month-old regenerated shoots were transferred onto RIM containing 3 mg/l IBA. All of the regenerated plants successfully induced roots in one week.

The regenerated potato plantlets from explants for microtuberization were cultured on the media MT-I and MT-II supplemented with 5% sucrose, IAA, kinetin and ascorbic acid. The tuberization rate of cultivars was shown in Table 3. The cvs. Astanalyk and Aksor cultured on MT-II medium produced a higher proportion of microtubers (33.3%) than on MT-I (27.2%), and they were white in color (Fig. 1F). The cvs. Tokhtar and Kunaev Monument failed to induce microtubers on either medium, although both had a good microtuberization ability during micropropagation.

**Table 3:** Response of culture media variant, number of tubers and tuberization rate. Number of tubers per shoots calculated by counting the plants that formed at least one tuber out of the total number of plants. Tuberization rate of cultivars calculated as the number of shoots with tubers divided by the total number of shoots. Each value represents mean ± SD of three replicated experiments.

Cultivar	Number of shoots	Number of tuber per shoots	Variant of MT media	MT rate (%)
Astanalyk	33	1.1 ± 0.09	MT-I	27.3
	31	1.3 ± 0.12	MT-II	33.3
Aksor	22	1.0 ± 0.12	MT-I	27.3
	27	1.2 ± 0.10	MT-II	33.3

## Discussion

Direct shoot regeneration has an advantage over indirect regeneration from callus since it allows maintenance of true-to-type plants and avoids somaclonal variations. Hence, in the current study, we evaluated direct shoot regeneration from four potato genotypes of Kazakhstan breeding using two different explants. Our results are in agreement with studies demonstrating that GA_3_ isan important plant hormone with the ability to induce a number of responses, including direct shoot regeneration (Ibragimova et al., 2018; Roest & Bokelmann, 1976), and suggesting that the combination of auxins and cytokinins in the presence of GA_3_ enhances shoot regeneration from explants (Campos et al., 2016; Kumlay, 2014).

Three factors, i.e., growth hormones, explant, and varieties, are known to have significantly different effects on direct shoot induction. Many shoots have been regenerated via the application of growth hormones. Various combinations of PGRs and explants for the regeneration of different potato cultivars have been reported (Anjum & Ali, 2004; Sarkar et al., 2015; Tara, Krishnaprasad & Veena, 2017). These reports are somewhat contradictory in terms of effectiveness in promoting shoot initiation and have shown zeatin to be less, equal, or often superior to BAP as the cytokinin component. Our data show that for direct shoot regeneration from local potato cultivars the combinations of zeatin and GA_3_ with IAA and NAA are more effective than the combination of BAP and GA_3_. These results are consistent with reports (Ibragimova et al., 2018; Manisha & Tapan, 2015) of using this combination of PGRs for other potato cultivars. The variable response of cultivars implies a vital role of genotypes in the direct shoot regeneration of potato in tissue culture.

Direct plant regeneration via multiple shoot induction is a preferred morphogenetic pathway for regeneration because it tends to produce genetically similar plants, as no callusogenesis is involved (Larkin & Scowcroft, 1981). However, the potato has a strong apical dominance, which inhibits the growth of multiple shoots from axillary buds (Levy, Seabrook & Coleman, 1993). It is known that optimum concentration and combination of growth hormones are crucial for multiple shoot formation in potato (Hoque, 2010; Mohapatra et al., 2018). Previously, it has been reported that kinetin at a concentration of 4 mg/l is the best cytokinin for multiple shoot regeneration (Hoque, 2010). Other studies (Manisha & Tapan, 2015; Shambhu, Hak & Hira, 2010) have suggested that the combined application of PGRs is more appropriate for direct shoot induction from internodal and nodal explants; moreover the raised plantlets produce microtubers in a period of 8–10 weeks.

Micropropagation of potato plants does not require the application of PGRs to rooting (Hussey & Stacey, 1981), whereas for root induction from the regenerated shoots of internode and leaf explants hormones are necessary (Mohapatra et al., 2018; Tara, Krishnaprasad & Veena, 2017). For instance, the roots were induced at a lower concentration of IBA (Mohapatra et al., 2018) and by a combination of NAA and GA_3_ at a concentration of 0.1 mg/l (Tara, Krishnaprasad & Veena, 2017).

in vitro tuberization in potato is influenced by many factors including genotype, PGRs and carbohydrate supply (Fischer et al., 2008; Hoque, 2010; Mohapatra et al., 2018). In the present work, green microtubers on axillary buds during micropropagation on a hormone-free MS medium containing 3% sucrose and microtuberization was achieved on the media MT-I and MT-II supplemented with 5% sucrose, although it is believed that increased sucrose concentration and PGRs are important for microtuberization and the production of larger-sized microtubers (Fischer et al., 2008; Levy, Seabrook & Coleman, 1993). Previously, a phenomenon like the induction of green microtubers on axillary buds was described in the ST mutant obtained by random gene activation (Fischer et al., 2008). The green colour might be due to the presence of alkaloids, such as solanine, produced under light conditions. The analysis of microtuberization potential of local potato cultivars presented here might be useful for research on modification of starch content.

## Conclusion

We report a wide response of potato cultivars Astanalyk, Monument Kunaev, Tokhtar, and Aksor on eight direct regeneration media differing in concentrations and combinations of PGRs. Our results clearly show that internode and leaf explants of the local potato cultivars are suitable for inducing direct multiple shoot regeneration. Significant differences were observed among cultivars and explants. Internode explants showed a higher frequency of shoot regeneration and a greater number of shoots per explant than leaf explants. The best medium for direct shoot regeneration was DRM-VIII supplemented with 0.1 mg/l IAA, 1.0 mg/l zeatin and 7.0 mg/l GA_3_, where cv. Astanalyk gave the highest shoot induction (90.0%) followed by Aksor (88%) and Tokhtar (70.0%). On this medium, the leaf explants of cvs. Aksor and Tokhtar yielded the best results, inducing 68% and 30.0% of shoots, respectively. The differences in the response of cultivars depending on the regeneration medium could be attributed to mature and more vascular tissue in the internodes and due to endogenous and exogenous hormones, which control the mode of morphogenetic response. Since the plantlets regenerated from the stem internode explants of cvs. Astanalyk and Aksor have the best microtuberization ability in tissue culture, these cultivars and the optimized tissue culture conditions will be used for developing genome-edited plants through direct shoot organogenesis. These results indicate that the comprehensive selection of PGRs for local potato cultivars is important for future tissue culture research creating opportunities for genetic improvement of potato cultivars.

##  Supplemental Information

10.7717/peerj.9447/supp-1 Supplemental Information 1 Raw data for Table 2

10.7717/peerj.9447/supp-2 Supplemental Information 2 Raw data indicates the effects of various growth regulators on the induction of multiple shoots from explants of the internodes of the stem varieties Astanalyk, Monument Kunaev, Tokhtar and Aksor, where the standard deviation and mean values are calculated that are used to analyze the data and prepare for the detailed study shown in Fig. 2.

10.7717/peerj.9447/supp-3 Supplemental Information 3 Raw data exported from a two-way ANOVA analysis of internode explants followed by a special Bonferroni test (^∗^*p* < 0.05) using Prism 5. The parametric method was used to compare four varieties that were compared with each other in each medium shown in Fig. 2.

10.7717/peerj.9447/supp-4 Supplemental Information 4 Raw data indicates the effects of various growth regulators on the induction of multiple shoots from explants of the leaves of the stem varieties Astanalyk, Monument Kunaev, Tokhtar and Aksor, where the standard deviation and mean values are calculated that are used to analyze the data and prepare for the detailed study shown in Fig. 3.

10.7717/peerj.9447/supp-5 Supplemental Information 5 Raw data exported from a two-way ANOVA analysis of leaf explants followed by a special Bonferroni test (^∗^*p* < 0.05) using Prism 5. The parametric method was used to compare four varieties that were compared with each other in each medium shown in Fig. 3.

## Additional Information and Declarations

### Data Availability

The following information was supplied regarding data availability: Primary statistical data and raw data are available in the Supplementary Files.
